# Whole‐brain atrophy assessed by proportional‐ versus registration‐based pipelines from 3T MRI in multiple sclerosis

**DOI:** 10.1002/brb3.1068

**Published:** 2018-07-18

**Authors:** Christopher C. Hemond, Renxin Chu, Subhash Tummala, Shahamat Tauhid, Brian C. Healy, Rohit Bakshi

**Affiliations:** ^1^ Laboratory for Neuroimaging Research Department of Neurology Brigham & Women's Hospital Partners MS Center Ann Romney Center for Neurologic Diseases Harvard Medical School Boston Massachusetts; ^2^ Laboratory for Neuroimaging Research Department of Radiology Brigham & Women's Hospital Partners MS Center Ann Romney Center for Neurologic Diseases Harvard Medical School Boston Massachusetts

**Keywords:** brain parenchymal fraction, brain parenchymal volume, multiple sclerosis, statistical parametric mapping, structural image evaluation using normalization of atrophy

## Abstract

**Background and Purpose:**

Whole‐brain atrophy is a standard outcome measure in multiple sclerosis (MS) clinical trials as assessed by various software tools. The effect of processing method on the validity of such data obtained from high‐resolution 3T MRI is not known. We compared two commonly used methods of quantifying whole‐brain atrophy.

**Methods:**

Three‐dimensional T1‐weighted and FLAIR images were obtained at 3T in MS (*n* = 61) and normal control (NC,* n* = 30) groups. Whole‐brain atrophy was assessed by two automated pipelines: (a) SPM8 to derive brain parenchymal fraction (BPF, proportional‐based method); (b) SIENAX to derive normalized brain parenchymal volume (BPV, registration method). We assessed agreement between BPF and BPV, as well their relationship to Expanded Disability Status Scale (EDSS) score, timed 25‐foot walk (T25FW), cognition, and cerebral T2 (FLAIR) lesion volume (T2LV).

**Results:**

Brain parenchymal fraction and BPV showed only partial agreement (*r* = 0.73) in the MS group, and *r* = 0.28 in NC. Both methods showed atrophy in MS versus NC (BPF *p* < 0.01, BPV *p* < 0.05). Within MS group comparisons, BPF (*p* < 0.05) but not BPV (*p* > 0.05) correlated with EDSS score. BPV (*p* = 0.03) but not BPF (*p* = 0.08) correlated with T25FW. Both metrics correlated with T2LV (*p* < 0.05) and cognitive subscales. BPF (*p* < 0.05) but not BPV (*p* > 0.05) showed lower brain volume in cognitively impaired (*n* = 23) versus cognitively preserved (*n* = 38) patients. However, direct comparisons of BPF and BPV sensitivities to atrophy and clinical correlations were not statistically significant.

**Conclusion:**

Whole‐brain atrophy metrics may not be interchangeable between proportional‐ and registration‐based automated pipelines from 3T MRI in patients with MS.

## 1 BACKGROUND

Whole‐brain atrophy is a commonly used research metric to quantify multiple sclerosis (MS) pathology (Neema, Stankiewicz, Arora, Guss, & Bakshi, 2007) and remains one of the strongest correlates and predictors of clinical status (De Stefano et al., 2014). Investigators have applied a myriad of published proprietary and open‐source methods to quantify brain volume loss (Giorgio, Battaglini, Smith, & De Stefano, 2008), leading to heterogeneous segmentation procedures across sites and studies, without any agreed‐upon standard approach (Bermel & Bakshi, 2006). This heterogeneity is brought to the surface by the regular incorporation of whole‐brain atrophy as a supportive outcome measure in Phase III MS therapeutic clinical trials, in which registration‐based {affine‐fit to an external multiple subject brain size atlas, e.g., normalized brain parenchymal volume [BPV; OPERA I/II (Hauser et al., 2017), FREEDOMS (De Stefano et al., 2016), ALLEGRO (Comi et al., 2012), DEFINE (Arnold et al., 2014)]}, or proportional‐based {scaled to the subject's own intracranial cavity, e.g., brain parenchymal fraction [BPF; CARE‐MS I/II (Arnold et al., 2016), AFFIRM (Miller et al., 2007), TEMSO (O'Connor et al., 2011)]} methods have been employed. Moreover, this challenge is amplified by the observations that the analysis of the same MRI image sets using different segmentation pipelines can produce conflicting findings (O'Connor et al., 2011; Radue et al., 2017; Rovaris, Comi, Rocca, Wolinsky, & Filippi, 2001; Sormani et al., 2004), which hamper the ability to draw firm conclusions on therapeutic effects, and may invalidate the comparison of results across trials.

Significant technical challenges arise in the measurement of cross‐sectional and longitudinal brain volume loss, especially at a fully automated scale necessary for efficient deployment in routine clinical practice. MRI‐derived volumetrics are prone to deviations throughout the data pipeline, including at the acquisition stage (e.g., head motion, hardware nonuniformity including magnetic field strength, gradient distortions, and pulse sequence type and parameters; Chu, Hurwitz, Tauhid, & Bakshi, 2017; Papinutto et al., 2017; Sharma et al., 2004; Shinohara et al., 2017) and segmentation procedure (e.g., preprocessing steps—inhomogeneity correction, method of tissue class segmentation, and normalization; Chard, Parker, Griffin, Thompson, & Miller, 2002; Chu, Hurwitz, et al., 2017; Durand‐Dubief et al., 2012; Granberg et al., 2016; Kazemi & Noorizadeh, 2014; Popescu, Schoonheim, et al., 2016; Vidal‐Jordana et al., 2017). Furthermore, brain volume may vary based on pathophysiological factors, including recent start of immunomodulatory therapy, acute inflammation, hydration status, time of day, tobacco use, genetics, and comorbid conditions (Rocca et al., 2017). As MRI technology evolves and increasingly precise high‐field (e.g., 3T) magnets proliferate in clinical practice, there remains an ongoing need for critical evaluation of the sensitivity and validity of postprocessing software pipelines (Chu et al., 2016; Stankiewicz et al., 2011).

Previous MRI research has explored methodological aspects of precision (i.e., reproducibility), accuracy (i.e., relation to gold standard maps), and validity (i.e., relationship to clinical “truth”) of whole‐brain and regional tissue loss in MS. Recent studies have examined the precision of metrics from 1.5T or 3T scanners using standardized acquisition parameters and software pipelines; all concluded that intrascanner variance was generally minimal, whereas interscanner variability was consistently a source of significant bias (Biberacher et al., 2016; Durand‐Dubief et al., 2012; Papinutto et al., 2017; Shinohara et al., 2017). The type of postprocessing software pipeline was also associated with divergent measurements in brain volumetrics in those studies. The accuracy and validity of MRI‐derived metrics has also been explored in reference to both clinical and histopathological metrics. A recent study by Popescu, Klaver, et al. (2016) correlated postmortem, histopathologically defined cortical thickness with MRI‐acquired cortical gray matter (GM) measurements at 1.5T; the authors found statistically significant correlations only when using manually corrected (but not automated) pipelines in SIENAX and FreeSurfer. A separate study from the same group compared postprocessing pipelines in SIENAX, SPM, and FreeSurfer to evaluate the link between GM atrophy and cognitive performance in MS; although the software pipelines generally exhibited similar clinical correlations with cognitive variables, the authors found significant differences in deep GM and cortical structure measurements based, at least partly, on the choice of registration template/atlas (Popescu, Schoonheim, et al., 2016). The goal of this study was to compare the validity of two freely available widely used automated postprocessing algorithms for the assessment of normalized whole‐brain volume from 3T MRI. We examined patients with MS and normal controls (NC) using two methods: both proportional‐based [SPM8 to measure BPF (Dell'Oglio et al., 2015)] and registration‐based (SIENAX to measure BPV).

## 2 METHODS

### 2.1 Subjects

We prospectively enrolled 61 patients with MS and 30 NC; part of the data from these subjects and the recruitment/collection procedures have been published previously (Dell'Oglio et al., 2015). In brief, inclusion criteria were: age 18–55, no significant medical comorbidities, no changes in disease‐modifying therapy in the 6 months prior to examination. MRI was obtained within 3 months of the neurological examination. Demographic and clinical data are summarized in Table 1. Clinical data were obtained by MS specialists, including Expanded Disability Status Scale (EDSS) scoring and timed 25‐foot walk (T25FW). This study was approved by our institutional board review board and all subjects provided written informed consent.

**Table 1:** Subject characteristics

	Multiple sclerosis	Normal controls
*N*	61	30
Age (years)	41.0 ± 8.6	43.9 ± 6.3
Women (*n*, %)	42 (69%)	21 (70%)
Disease duration (years since first symptoms)	8.3 ± 7.2	–
EDSS score	1.6 ± 1.7	–
Timed 25‐foot walk (seconds)	4.8 ± 4.5	–
T2 (FLAIR) hyperintense lesion volume (ml)	13.6 ± 11.4	0.44 ± 0.57
Clinical disease category (*n*, %)		
Clinically isolated syndrome	4 (6.6%)	–
Relapsing–remitting	51 (83.6%)	–
Secondary progressive	5 (8.2%)	–
Primary progressive	1 (1.6%)	–
Receiving disease‐modifying therapy (*n*, %)	49 (80.3%)	–
MACFIMS‐classified cognitive impairment (*n*, %)	23 (37.7%)	–

*Notes*

Data are shown as mean ± *SD* unless otherwise noted.

EDSS: Expanded Disability Status Scale; FLAIR: fluid‐attenuated inversion recovery; MACFIMS: Minimal Assessment of Cognitive Function in MS; *n*: number of subjects; group comparison: Age, *p* = 0.10 (two‐sample *t* test), gender, *p* = 0.91 (Pearson's χ^2^).

### 2.2 Neuropsychological data acquisition and analysis

Complete neuropsychological evaluation methods are described previously (Dell'Oglio et al., 2015). In brief, this was based on the previously validated Minimal Assessment of Cognitive Function in MS (MACFIMS) battery (Benedict et al., 2006), which was administered by a clinical psychologist and her supervised research fellow. MACFIMS scores were corrected for depression (CES‐D) baseline scores, and compared to regression‐based norms from a NC sample (Parmenter, Testa, Schretlen, Weinstock‐Guttman, & Benedict, 2010). Cognitive impairment was defined as performance worse than the 5th percentile on two or more cognitive measures; subjects who did not meet these criteria were defined as cognitively preserved.

### 2.3 MRI acquisition

All subjects were scanned on the same 3T MRI unit (Signa; General Electric, Milwaukee, WI, USA) using an identical brain acquisition protocol among all subjects: coronal 3D modified driven equilibrium Fourier transform (MDEFT) covering the whole head: TR = 7.9 ms, TE = 3.14 ms, flip angle = 15°, slice thickness = 1.6 mm, pixel size = 0.938 × 0.938 mm; axial 2D T2‐weighted fast fluid‐attenuated inversion recovery (FLAIR): TR = 9,000 ms, TE = 151 ms, TI = 2,250 ms, slice thickness = 2 mm (no gap), matrix size = 256 × 256, pixel size = 0.976 × 0.976 mm.

### 2.4 Image analysis

**Figure 1:**
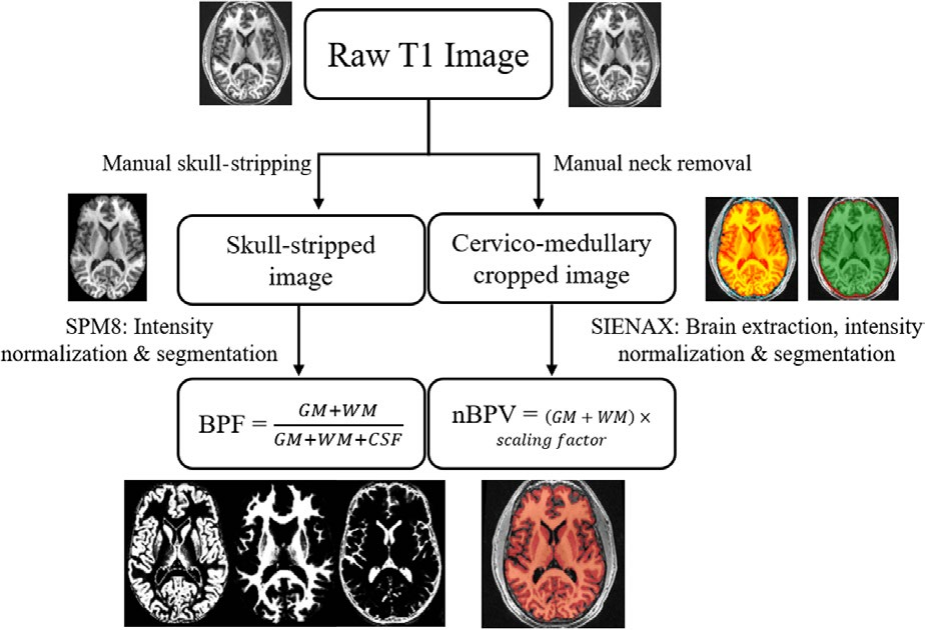
Comparison of image processing steps for the proportional‐ and registration‐based methods of determining normalized whole‐brain volume. Both methods used the same 3D, T1‐weighted MDEFT source images at 3T. Brain parenchymal fraction (BPF, left, a proportional‐based method) began with manual skull‐stripping, followed by automated SPM8 registration to the MNI‐152 atlas, nonparametric intensity normalization, and tissue class segmentation with bias field tool disabled, yielding mutually exclusive maps for CSF (cerebrospinal fluid), gray matter (GM), and white matter (WM). BPF (bottom left) is calculated as the sum of the gray and white matter volumes divided by the total intracranial volume represented as the sum of GM + WM + CSF. Normalized brain parenchymal volume (BPV, right, a registration‐based method), began with manual neck removal to the cervico‐medullary junction, followed by automated SIENAX‐based brain extraction with bias field correction enabled (orange highlight), registration to the MNI‐152 template to determine the skull‐based scaling factor, and intensity normalization and tissue class segmentation using a Markov random field model with the associated expectation‐maximization algorithm. GM and WM volumes are summated to yield the BPV, which is multiplied by a subject‐specific scaling factor to yield normalized BPV (red highlight, bottom right)

All images were inspected for quality, and processed through two separate pipelines (Figure 1); BPF: as previously described (Dell'Oglio et al., 2015), raw MDEFT images were manually de‐skulled, aligned to the MNI152 template, intensity normalized using N3 nonparametric nonuniform parameters, and automatically segmented using the SPM8 (Statistical Parametric Mapping, http://www.fil.ion.ucl.ac.uk/spm/software/) unified segmentation model into GM, white matter (WM), and CSF volumes. Intracranial volume (ICV) was calculated as the sum of GM + WM + CSF. BPF was calculated as (GM + WM)/ICV. In the BPV pipeline, raw MDEFT images were resliced to the axial plane, followed by removal of all slices inferior to the cervico‐medullary junction using JIM v7 (http://www.xinapse.com). Images then underwent automated segmentation and template normalization using SIENAX, (Smith et al., 2002) part of FSL (v5.0) (Smith et al., 2004) using a previously optimized brain extraction tool (BET) threshold of 0.2 (Chu et al., 2016). T2‐hyperintense lesion volumes were obtained by expert semiautomated segmentation with an edge‐finding tool based on local image intensity thresholds using JIM (v5) as previously published (Dell'Oglio et al., 2015); manual corrections were applied as needed (Ceccarelli et al., 2012). To determine if manual versus default (automated) deskulling would affect the results in SIENAX, we analyzed scans from three subjects using manually skull‐stripped images with a BET threshold of 0.01 (for maximal brain extraction) normalized with the original scaling factor from nonskull‐stripped data; however, this approach provided similar BPVs (within 20 ml versus the nonskull‐stripped extraction, mean ± *SD* = −5.66 ± 22.2, range: −19 to 20 ml). Thus, we chose to employ the fully automated SIENAX algorithm to obtain BPV in this study.

### 2.5 Statistical analysis

Correlations between BPF and BPV in MS and NC populations were calculated using Pearson's correlation coefficients. Group differences between MS and NC were calculated using *t* tests, and linear regression to correct for age and gender. The difference in the estimated effect size comparing MS and NC for the BPF and BPV segmentation methods was calculated as the difference in Cohen's d and the 95% confidence interval (CI) for the difference was calculated using the percentile bootstrap method. Clinical correlations were obtained using Spearman's correlation coefficient (EDSS, T25FW, disease duration) and Pearson's correlation coefficient (age, gender), and partial correlation coefficients were used to correct for age and gender. For the comparison between the BPF and BPV segmentation methods regarding their correlations with EDSS and T2FW, the difference in the correlation coefficients was calculated, and the 95% CI was calculated using the percentile bootstrap method. Correlations between BPF, BPV, and components of the MACFIMS were estimated using Pearson's correlation coefficients and partial correlation coefficients were used to adjust for age and gender. In addition, Meng's test was used to compare the correlated correlation coefficients between the BPF and BPV measurements and the MACFIMS components (Meng, Rosenthal, & Rubin, 1992). *p*‐Values <0.05 were considered statistically significant. Analyses were performed using the R software (http://www.r-project.org) with the pcor (Kim, 2015) and cocor (Diedenhofen & Musch, 2015) libraries.

## 3 RESULTS

**Figure 2:**
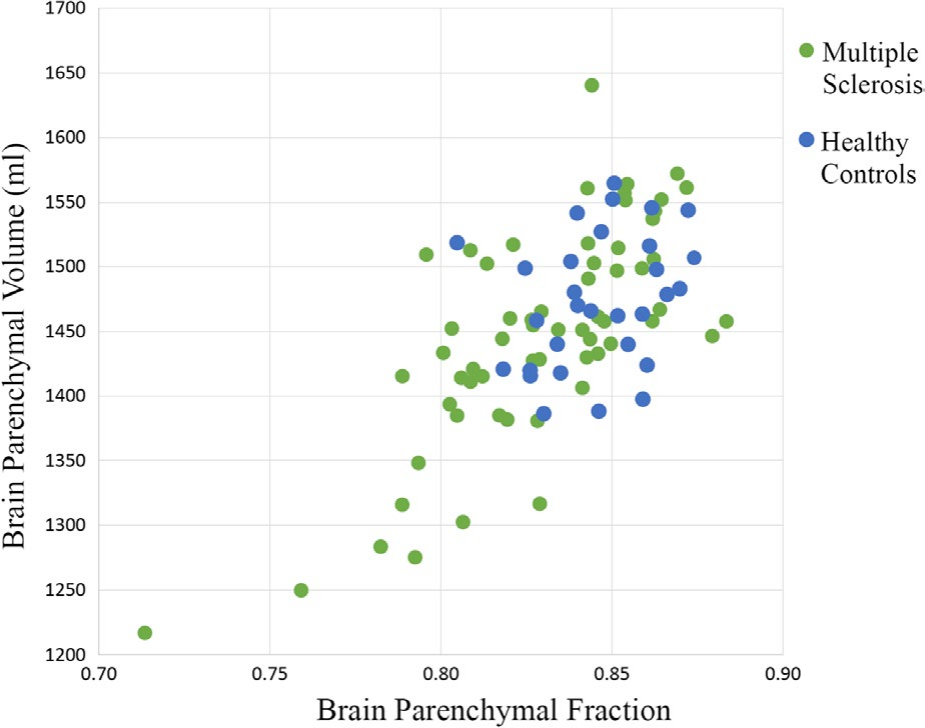
Scatterplot between two normalized measures of whole‐brain volume. In all subjects, *r* = 0.671, *p* < 0.001. Within individual groups: multiple sclerosis *r* = 0.725, *p* < 0.001; normal controls *r* = 0.13, *p* = 0.137. Results are Pearson's correlation *r*

**Figure 3:**
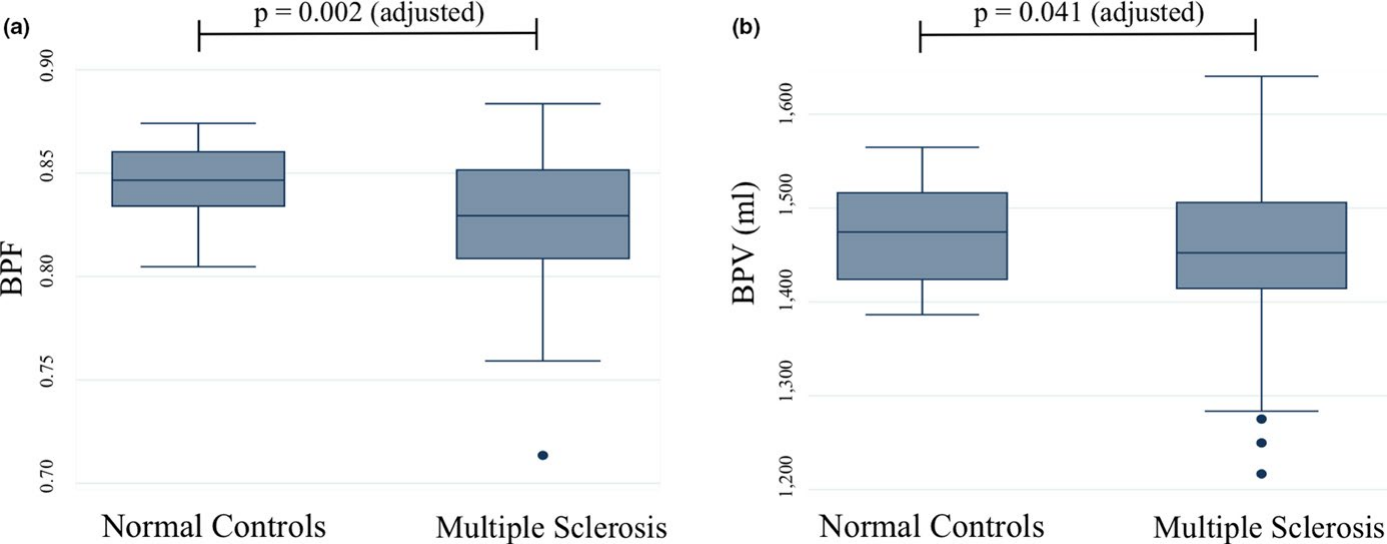
Comparing two measures of normalized whole‐brain volume between groups. Box‐and‐whisker plots shown with interquartile ranges (box), next adjacent values (whiskers) and outliers notated as points. Unadjusted *p*‐values were determined by *t* tests; adjusted *p*‐values determined by linear regression after adjustment for age and gender. (a) BPF = brain parenchymal fraction, unadjusted means: BPF (normal control) = 0.846 ± 0.017, BPF (multiple sclerosis) = 0.830 ± 0.031, *p* = 0.002; adjusted *p* = 0.002 (b) BPV = normalized brain parenchymal volume, unadjusted means: BPV (normal controls) = 1,474.4 ± 51.7 ml, BPV (multiple sclerosis) = 1,448.4 ± 84.2 ml, *p* = 0.073, adjusted *p* = 0.041

The correlation between BPF and BPV is shown in Figure 2. The two measures showed a moderate intercorrelation across all subjects (*r* = 0.671, *p* < 0.001). The correlation varied between groups (MS, *r* = 0.725, *p* < 0.001; NC, *r* = 0.278, *p* = 0.137). Group differences in the normalized whole‐brain volume measures are shown in Figure 3. The unadjusted comparison indicated that BPF showed whole‐brain atrophy in the MS versus NC group (*p* = 0.002), which remained significant after correction for age and gender (*p* = 0.002); BPV showed whole‐brain atrophy in the MS versus NC group following adjustment for age and gender (*p* = 0.041), but not in the unadjusted comparison (*p* = 0.073). Effect sizes for discriminating MS and NC groups did not differ in direction comparisons between BPF and BPV (95% CI: −0.643, 0.113, *p* > 0.05). Regarding the correlations between BPF or BPV and neurologic function/lesion variables (Table 2), BPF negatively correlated with disease duration (*r* = −0.338, *p* = 0.008), EDSS (*r* = −0.290, *p* = 0.023), and T2LV (*r* = −0.305, *p* = 0.017) prior to correction for age and gender; following correction these comparisons remained significant except disease duration. By comparison, BPV typically did not attain statistical significance in these correlations; after correction for age and gender, significance was attained with T25FW and T2LV measures only. However, the differences in correlation with disability between the segmentation methods were not statistically significant. The relationship between normalized whole‐brain volume measures and cognition is shown in Tables 3 and 4.

**Table 2:** Two normalized whole‐brain volume measures correlated with clinical/lesion variables in the MS group (*n* = 61)

	Unadjusted		Adjusted	
	BPF	BPV	BPF	BPV
Age	−0.322 (0.011)^a^	−0.333 (0.009)^a^	−0.262 (0.043)^a^	−0.319 (0.013)^a^
Disease duration	−0.338 (0.008)^a^	−0.214 (0.097)	−0.246 (0.060)	−0.058 (0.663)
EDSS	−0.289 (0.024)^a^	−0.223 (0.083)	−0.264 (0.043)^a^	−0.188 (0.154)
T25FW	−0.171 (0.187)	−0.225 (0.081)	−0.207 (0.116)	−0.286 (0.028)^a^
T2LV	−0.305 (0.017)^a^	−0.274 (0.033)^a^	−0.318 (0.014)^a^	−0.291 (0.025)^a^

*Notes*

Age and disease duration results are Pearson's correlation *r*, (*p*‐value); EDSS, T25FW, T2LV results are Spearman correlation *r*, (*p*‐value). Following adjustment for age and gender, the results provided are partial correlations; age is corrected for gender only.

BPF: brain parenchymal fraction; BPV: normalized brain parenchymal volume; EDSS: Expanded Disability Status Scale; T25FW: timed 25‐foot walk; T2LV: cerebral T2 hyperintense lesion volume; *n*: number of subjects.

a *p *<* *0.05.

**Table 3:** Two normalized whole‐brain volume measures: relationship to cognitive status in the MS group

	Cognitively impaired (*n* = 23)	Cognitively preserved (*n* = 38)	*p*‐Value (unadjusted)	*p*‐Value (adjusted)
BPF	0.817 ± 0.034	0.837 ± 0.026	0.024*	0.033*
BPV (ml)	1,422.3 ± 91.4	1,464.2 ± 76.6	0.073	0.139

*Notes*

Values are mean ± *SD*.

BPF: brain parenchymal fraction; BPV: normalized brain parenchymal volume; *n*: number of subjects.

Unadjusted *p*‐values determined by *t* tests; adjusted *p*‐values determined by linear regression after adjustment for age and gender. Cognitive impairment threshold based on Minimal Assessment of Cognitive Function in multiple sclerosis scores: *p < 0.05.

**Table 4:** Two normalized whole‐brain volume measures: correlation with cognitive component scores in the MS group (*n* = 61)

	Unadjusted		Adjusted	
	BPF	BPV	BPF	BPV
PASAT3	0.227 (0.079)	0.213 (0.100)	0.247 (0.059)	0.235 (0.074)
COWAT	0.129 (0.323)	0.121 (0.352)	0.305 (0.019)^a^	0.318 (0.014)^a^
BVMT TL	0.128 (0.325)	0.034 (0.797)	0.109 (0.410)	0.005 (0.967)
BVMT DR	0.215 (0.096)	0.126 (0.335)	0.226 (0.086)	0.131 (0.324)
JLO	0.264 (0.039)^a^	0.237 (0.066)	0.336 (0.009)^a^	0.300 (0.021)^a^
SDMT	0.375 (0.003)^a^	0.301 (0.018)^a^	0.363 (0.005)^a^	0.273 (0.036)^a^
CVLT TL	0.211 (0.103)	0.203 (0.116)	0.281 (0.031)^a^	0.282 (0.030)^a^
CVLT DR	0.262 (0.042)^a^	0.212 (0.101)	0.247 (0.059)	0.196 (0.136)
DKEFS CS	0.103 (0.427)	0.082 (0.530)	0.166 (0.209)	0.154 (0.243)
DKEFS DS	0.142 (0.274)	0.127 (0.329)	0.149 (0.260)	0.141 (0.287)

*Notes*

All data are Spearman correlation *r* (unadjusted values) or partial Spearman correlation *r* (adjusted for age and gender), followed by *p*‐values in parentheses. For all cognitive tests, we used the T scores from the regression‐based norms.

BPF: brain parenchymal fraction; BPV: brain parenchymal volume; BVMT: Brief Visuospatial Memory Test (TL: total recall; DR: delayed recall); COWAT: Controlled Oral Word Association Test; CVLT: California Verbal Learning Test (TL: 5‐trial recall, DR: delayed recall); DKEFS: Delis–Kaplan Executive Function System Test (CS: total confirmed correct sorts, DS: total description score); JLO: Judgment of Line Orientation test; MACFIMS: Minimal Assessment of Cognitive Function in Multiple Sclerosis; *n*: number of subjects; PASAT3: Paced Auditory Serial Addition Task (3‐s delay); SDMT: Symbol Digit Modalities Test.

a *p* < 0.05.

Brain parenchymal fraction showed statistically significant differences in whole‐brain volume in cognitively impaired versus cognitively preserved patients both before (*p* = 0.02) and after (*p* = 0.03) age and gender correction (Table 3). However, there was only a trend toward lower whole‐brain volume as measured by BPV in cognitively impaired versus cognitively preserved patients (*p* = 0.073), which did not attain significance following adjustment for age and gender (*p* = 0.14, Table 3). Table 4 shows the correlations between the normalized whole‐brain volume measures and cognitive (MACFIMS) subsets. Both BPF and BPV were significantly correlated with the Symbol Digit Modalities Test scores in unadjusted comparisons and following adjustment for age and gender. In addition, both BPF and BPV significantly correlated with scores on the Controlled Oral Words Association Test, Judgment of Line Orientation, and California Verbal Learning Test. The directions of the correlations indicated that whole‐brain atrophy was associated with higher disease severity. A comparison of the two segmentation methods for their correlations with cognitive test variables did not reveal any significant differences (*p* > 0.3 for all cognitive tests).

## 4 DISCUSSION

Our cross‐sectional study suggests a difference in whole‐brain volume measures obtained from two postprocessing pipelines from 3T MRI high‐resolution 3D T1‐weighted images in people with MS, apparent from several perspectives. First, we have shown that data from the two pipelines are only moderately intercorrelated in MS and weakly correlated in NC. This discrepancy in correlations was unexpected; we speculate it may be related to improved algorithmic selection of the brain–CSF interfaces in the MS group, who have greater overall atrophy and therefore more CSF space around the brain parenchymal and sulcal folds; similarly, González‐Villà et al. (2017) found occasionally improved segmentation results in MS patients compared to controls. Second, BPF showed generally stronger associations as assessed by the detection of brain atrophy in patients with MS versus NC, MRI–clinical correlations with disability measures in the MS group, and the association of brain atrophy with cognitive impairment in the MS group. However, the differences between the pipelines in their clinical associations was not robust and did not reach statistical significance in direct comparisons of the two methods, perhaps related to the sample size. In addition, our T1‐weighted 3D MDEFT sequence did not use isotropic voxel sizes which may have decreased precision. However, it should be noted that this sequenced showed excellent scan–rescan reproducibility in a separate study of 11 subjects for the assessment of normalized whole‐brain and regional deep gray matter volume (mean coefficient of variation <1%; Chu, Kim et al., 2017).

Our data demonstrate that cross‐sectional postprocessing methods require careful interpretation, especially as brain volume loss evolves into a potential metric for clinical decision‐making in MS. Our results are in line with several prior studies which have demonstrated improved MS‐related clinical validity for a proportion‐based over a registration‐based metric for cross‐sectional data. Gao and colleagues used a heavily T2‐weighted approach at 3T to determine the total volume of intracranial cerebrospinal fluid and derived a “brain free water” fraction similar to (inverse) BPF; this parameter outperformed a T1‐weighted registration‐based approach (Lesion‐TOADS) correlating with clinical variables including EDSS score, the 9‐hole peg test, and the symbol digit modalities test (Gao, Nair, Cortese, Koretsky, & Reich, 2014). A separate group found that BPF derived from semiautomated methods at 1.5T outperformed the automated registration‐based method using SIENAX in regards to accuracy and clinical validity with EDSS (Zivadinov et al., 2005), although this could be at least partially attributed to suboptimal brain extraction with the latter method.

Comparisons of postprocessing pipelines are complicated by the sheer number of potential underlying variables that differ between methods, as well as a lack of a clear “ground truth” gold standard. Here we chose a pragmatic high‐level approach to compare pipeline clinical validity; other authors have previously compared individual processing steps as well, yielding insight into sources of variability in healthy populations or simulated datasets. The SPM and FSL pipelines used here rely on inherently different statistical models and assumptions when performing (a) brain extraction, (b) intensity normalization and tissue segmentation, and (c) template registration/normalization. Thus, one potential limitation of our study is that we cannot specify contributions of each of these factors to overall errors in clinical validity. Regarding (a) brain extraction, our BPF pipeline employed manually skull‐stripped data whereas our BPV pipeline used native images as generally required to obtain a skull‐based normalization factor. Although manual skull‐stripping is closer to a gold standard for determining ICV, it is time‐consuming and has been largely replaced with automated techniques such as BET (Smith et al., 2002), SPM's integrated tissue segmentation (Ashburner & Friston, 2005), or FreeSurfer watershed algorithm (Dale et al., 2004). As prior authors have noted, the FSL BET can also be a significant source of error (Popescu et al., 2012; Zivadinov et al., 2005) and we found tissue misclassification in several subjects using the default settings; neck cropping and changing the default parameters (−f 0.2 and −B enabled) allowed an optimal solution for our dataset without any significant misclassification errors (Chu et al., 2016). Without any visually prominent errors, several groups have concluded that brain extraction methods are generally a very small source of variance (Clark, Woods, Rottenberg, Toga, & Mazziotta, 2006; Klauschen, Goldman, Barra, Meyer‐Lindenberg, & Lundervold, 2009) and we feel this preprocessing step is unlikely to be a significant source of variance between methods.

Regarding intensity normalization and tissue segmentation, both SPM and SIENAX use an integrated approach to this process (Ashburner & Friston, 2005; Smith et al., 2004; Zhang, Brady, & Smith, 2001). One advantage to using whole‐brain atrophy as a metric is its relative insensitivity to GM and WM tissue misclassification, as these two measures are summated to yield whole‐brain volumes. Tissue misclassification problems are exacerbated in MS due to T1‐hypointense lesions, which are shown to bias automated measures of gray versus white matter segmentation (Battaglini, Jenkinson, & De Stefano, 2012; Ceccarelli et al., 2012). Although lesion filling techniques improve accuracy of tissue segmentation (Chard, Jackson, Miller, & Wheeler‐Kingshott, 2010; González‐Villà et al., 2017), they did not appear to substantially affect measures of whole‐brain volume as we chose to use here (Dell'Oglio et al., 2015). There is an extensive literature regarding the optimization of GM versus WM tissue segmentation postprocessing techniques (Derakhshan et al., 2010; Popescu, Schoonheim, et al., 2016; Rocca et al., 2017), which is beyond the scope of this paper.

A third potentially important difference between our pipelines is the template registration and normalization process. Whereas the BPF metric normalizes brain volume using the subject's own intracranial volume, BPV normalizes to a registered template (MNI‐152) of averaged healthy brains. We speculate that this difference in normalization factor may help explain why a proportion‐based metric may be superior to a registration‐based metric regarding clinical validity. This topic has not received significant attention in the literature and would be worth exploring in more detail in future experiments with longitudinal comparisons.

## 5 CONCLUSION

Determination of whole‐brain atrophy on 3T MRI depends in part on the choice of postprocessing software methods; here, a comparison of automated pipelines revealed discrepant results for whole‐brain atrophy measures and clinical correlations, likely based on the underlying statistical assumptions for tissue segmentation and scaling methods of the software. Results obtained using these automated pipelines are unlikely to be interchangeable and should therefore be interpreted with caution.
